# Rhodium-Catalyzed Linear Codimerization and Cycloaddition of Ketenes with Alkynes

**DOI:** 10.3390/molecules15064189

**Published:** 2010-06-09

**Authors:** Teruyuki Kondo, Masatsugu Niimi, Yuki Yoshida, Kenji Wada, Take-aki Mitsudo, Yu Kimura, Akio Toshimitsu

**Affiliations:** 1 Advanced Biomedical Engineering Research Unit, Kyoto University, Katsura, Nishikyo-ku, Kyoto 615-8510, Japan; E-Mail: ykimura@scl.kyoto-u.ac.jp (Y.K.); 2 Department of Energy and Hydrocarbon Chemistry, Graduate School of Engineering, Kyoto University, Katsura, Nishikyo-ku, Kyoto 615-8510, Japan;E-Mails: Masatsugu_Niimi@jsr.co.jp (M.N.); yoshida.y@ky3.ecs.kyoto-u.ac.jp (Y.Y.); wadaken@scl.kyoto-u.ac.jp (K.W.); mitsudo@tech.email.ne.jp (T.M.); 3 Office of Society-Academia Collaboration for Innovation, Kyoto University, Yoshida-Honmachi, Sakyo-ku, Kyoto 606-8501, Japan; E-Mail: akiot@saci.kyoto-u.ac.jp (A.T.)

**Keywords:** rhodium, catalyst, ketene, alkyne, codimerization, cycloaddition

## Abstract

A novel rhodium-catalyzed linear codimerization of *alkyl phenyl ketenes* with internal alkynes to dienones and a novel synthesis of furans by an unusual cycloaddition of *diaryl ketenes* with internal alkynes have been developed. These reactions proceed smoothly with the same rhodium catalyst, RhCl(PPh_3_)_3_, and are highly dependent on the structure and reactivity of the starting ketenes.

## 1. Introduction

Ketenes are very important intermediates in the field of organic synthesis [1,2,3], and much attention has been focused on the ketene-metal complexes [4]. In general, ketenes coordinate to transition-metal complexes in two ways: 1) coordination through a C=C bond in ketenes [5], and 2) coordination through a C=O bond in ketenes [6,7,8,9,10]. If these coordination modes can be controlled through the selection of ketenes in transition-metal catalysis, completely different methods for the construction of novel organic molecules could be developed according to the structure and reactivity of ketenes using the same transition-metal catalyst.

We have previously developed a ruthenium-catalyzed synthesis of pyranopyrandiones by the ring-opening carbonylation of cyclopropenones [11] and a novel synthesis of 2-pyranones by the ruthenium- or rhodium catalyzed ring-opening dimerization of cyclobutenones [12], as well as a rhodium-catalyzed synthesis of 2-substituted phenols from cyclobutenones and alkenes via cleavage of a carbon-carbon bond [13]. We propose that (*η*^4^-bisketene)- and (*η*^4^-vinylketene)metal complexes are important key intermediates in these reactions; however, there are still few examples of transition-metal complex-catalyzed transformations of ketenes *themselves* [14,15,16,17,18,19,20]. Thus, we focused our attention on the development of novel reactions of ketenes with unsaturated compounds in the presence of ruthenium or rhodium catalysts, and recently developed rhodium-catalyzed decarbonylative coupling reactions of diphenyl ketene with 2-norbornenes and electron-deficient alkenes [21]. Then, the reactions of ketenes with alkynes were investigated in the presence of several transition-metal catalysts. After many trials, we developed the novel RhCl(PPh_3_)_3_-catalyzed linear codimerization of *alkyl phenyl ketenes* with internal alkynes and a novel synthesis of furans by the unusual RhCl(PPh_3_)_3_-catalyzed cycloaddition of *diaryl ketenes* with internal alkynes. In these reactions, the catalyst is the same but the products are completely different, depending on the structure and reactivity of the starting ketenes.

## 2. Results and Discussion

**Scheme 1:**
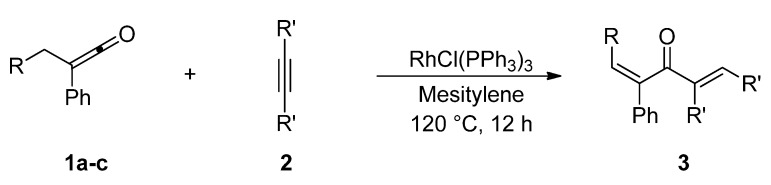
Rhodium-catalyzed linear codimerization of ketenes with alkynes to dienones.

Treatment of *alkyl phenyl ketenes ***1a**-**c **with internal alkynes **2** in the presence of 5 mol % RhCl(PPh_3_)_3_ in mesitylene at 120 ºC for 12 h under an argon atmosphere gave the corresponding dienones **3** in high yield with high stereoselectivity (Scheme 1). For example, RhCl(PPh_3_)_3_-catalyzed reaction of ethyl phenyl ketene (**1a**) with 3-hexyne (**2a**) gave only (2*Z*, 5*E*)-5-ethyl-3-phenylocta-2,5-dien-4-one (**3a**) in 92% yield, and no stereoisomers were obtained at all. 

First, the catalytic activities of several transition-metal complexes were examined in the reaction of **1a** with **2a**, and the results are summarized in Table 1. Among the catalysts examined, RhCl(PPh_3_)_3_ (**3a**, 92%) showed the highest catalytic activity. RhCl(CO)(PPh_3_)_2_ (**3a**, 46%) and RhCl_3_^.^3H_2_O (**3a**, 26%) also showed moderate catalytic activity; however, other rhodium complexes, such as RhH(PPh_3_)_4_ and RhH(CO)(PPh_3_)_3_, as well as ruthenium complexes, such as RuCl_2_(PPh_3_)_3_, [RuCl_2_(CO)_3_]_2_, and RuH_2_(PPh_3_)_4_, and an iridium complex, IrCl(CO)(PPh_3_)_2_, were totally ineffective, whereas Pd(PPh_3_)_4_ showed slight catalytic activity (**3a**, 29%). An attempt to reduce the amount of RhCl(PPh_3_)_3_ catalyst from 5.0 mol % to 2.0 mol % resulted in vain (Entry 2). 

**Table 1:** Catalytic activity of several transition-metal complexes in the reaction of **1a** with **2a **to **3a**.

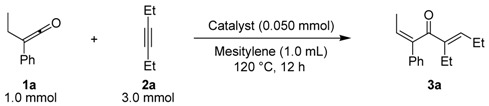					
Entry	Catalyst	Yield of 3a (%) ^a^	Entry	Catalyst	Yield of 3a (%) ^a^
1	RhCl(PPh_3_)_3_­	92	7	RuCl_2_(PPh_3_)_3_	1
2 ^b^	RhCl(PPh_3_)_3_	5	8 ^c^	[RuCl_2_(CO)_3_]_2_	0
3	RhCl(CO)(PPh_3_)_2_	46	9	RuH_2_(PPh_3_)_4_	0
4	RhCl_3_·3H_2_O	26	10	Pd(PPh_3_)_4_	29
5	RhH(PPh_3_)_4_	2	11	IrCl(CO)(PPh_3_)_2_	0
6	RhH(CO)(PPh_3_)_3_	0			
^a^ GLC yield; ^b^ RhCl(PPh_3_)_3_ (2.0 mol %, 0.020 mmol) for 40 h; ^c^ [RuCl_2_(CO)_3_]_2_ (0.025 mmol).					

**Table 2:** RhCl(PPh_3_)_3_-catalyzed linear codimerization of alkyl phenylketenes **1a**-**c** with internal alkynes **2a**,**b** to dienones **3a**-**d**.^a^

Entry	Ketene		Alkyne	Product		Isolated Yield (%)
1	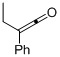	**1a**	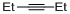	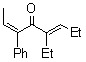	**3a**	74 (92) ^b^
			**2a**			
2	**1a**		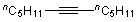	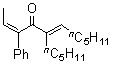	**3b**	68
			**2b**			
3	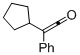	**1b**	**2a**	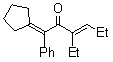	**3c**	52
4	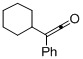	**1c**	**2a**	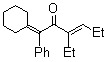	**3d**	40
^a^ Ketene (1.0 mmol), alkyne (3.0 mmol), RhCl(PPh_3_)_3_ (0.050 mmol), and mesitylene (1.0 mL) at 120 ºC for 12 h under an argon atmosphere; ^b^ GLC yield.						

The results obtained in the reaction of several *alkyl phenyl ketenes*** 1a**-**c** with alkynes **2a** and **b** under the optimized reaction conditions are summarized in Table 2. Ethyl phenyl ketene (**1a**) reacted with 6-dodecyne (**2b**) to give the corresponding dienone **3b** in an isolated yield of 68% (Entry 2). As for ketenes, cycloalkyl phenyl ketenes, such as **1b** and **1c**, also reacted with **2a** to give the corresponding dienones, **3c** and **3d**, in isolated yields of 52% and 40%, respectively (Entries 3 and 4). Unfortunately, when terminal alkynes, such as phenylacetylene and 1-hexyne, were used in RhCl(PPh_3_)_3_-catalyzed reaction with ethyl phenyl ketene (**1a**), the corresponding dienones were obtained in low yield (below 10%) together with various byproducts, probably due to the formation of a (vinylidene)Rh species.

**Scheme 2:**
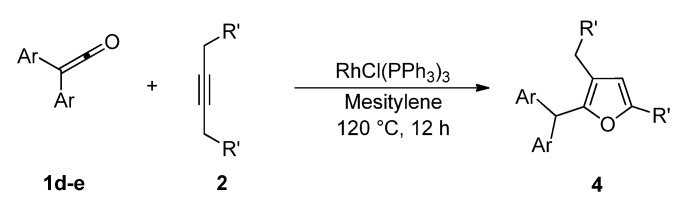
Rhodium-catalyzed cycloaddition of ketenes with alkynes to furans.

In sharp contrast, treatment of *diaryl ketenes ***1d** and **e** instead of *alkyl phenyl ketenes ***1a-c** with internal alkynes **2** in the presence of the same RhCl(PPh_3_)_3_ catalyst (5 mol %) in mesitylene at 120 ºC for 12 h under an argon atmosphere gave unusual cycloadducts, the furans **4**, instead of dienones **3**, in good to high yields (Scheme 2). The structure of furan **4a** was confirmed by ^13^C Inadequate NMR measurement (see Experimental, Figure 2).

The catalytic activities of several transition-metal complexes were also examined in the reaction of diphenyl ketene (**1d**) with 3-hexyne (**2a**), and the results are summarized in Table 3. Among the catalysts examined, only RhCl(PPh_3_)_3_ showed catalytic activity (**4a**, 74%). Other rhodium, ruthenium, iridium and palladium complexes were totally ineffective in the present reaction.

**Table 3:** Catalytic activity of several trasition-metal complexes in the reaction of **1d** with **2a** to **4a**.

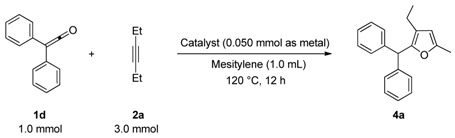					
Entry	Catalyst	Yield of 4a (%) ^a^	Entry	Catalyst	Yield of 4a (%) ^a^
1	RhCl(PPh_3_)_3_­	74	7	[Cp*RuCl_2_]_2_	0
2	RhCl(CO)(PPh_3_)_2_	2	8	RuCl_2_(PPh_3_)_3_	0
3	RhCl_3_·3H_2_O	0	9	[RuCl_2_(CO)_3_]_2_	0
4	[RhCl(cod)]_2_	0	10	IrCl(CO)(PPh_3_)_2_	0
5	[RhCl(C_2_H_4_)_2_]_2_	0	11	Pd(PPh_3_)_4_	0
6	RhH(PPh_3_)_4_	0			
^a^ GLC yield.					

6-Dodecyne (**2b**), as well as 4-octyne (**2c**) and 5-decyne (**2d**), reacted with **1d** to give the corresponding furans **4b-d** in moderate yields (Entries 2-4 in Table 4). As for ketenes, di(4-chloro-phenyl) ketene (**1e**) also reacted with **2a** to give the corresponding furan **4e** in an isolated yield of 51% (Entry 5).

**Table 4:** RhCl(PPh_3_)_3_-catalyzed unusual cycloaddition of diaryl ketenes **1d**, **e** with internal alkynes **2a**-**d** to furans **4a**-**e**.^a^

Entry	Ketene			Alkyne	Product		Isolated Yield (%)
1	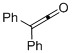	**1d**		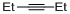	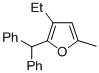	**4a**	64 (74) ^b^
				**2a**			
2	**1d**			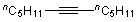	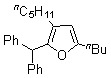	**4b**	43
				**2b**			
3	**1d**			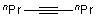	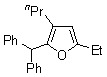	**4c**	52
				**2c**			
4	**1d**			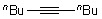	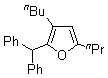	**4d**	43
				**2d**			
5	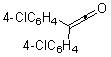		**1e**	**2a**	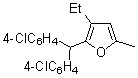	**4e**	51 (70)^b^
^a^ Ketene (1.0 mmol), alkyne (3.0 mmol), RhCl(PPh_3_)_3_ (0.050 mmol), and mesitylene (1.0 mL) at 120 ºC for 12 h under an argon atmosphere; ^b^ GLC yield.							

**Scheme 3:**
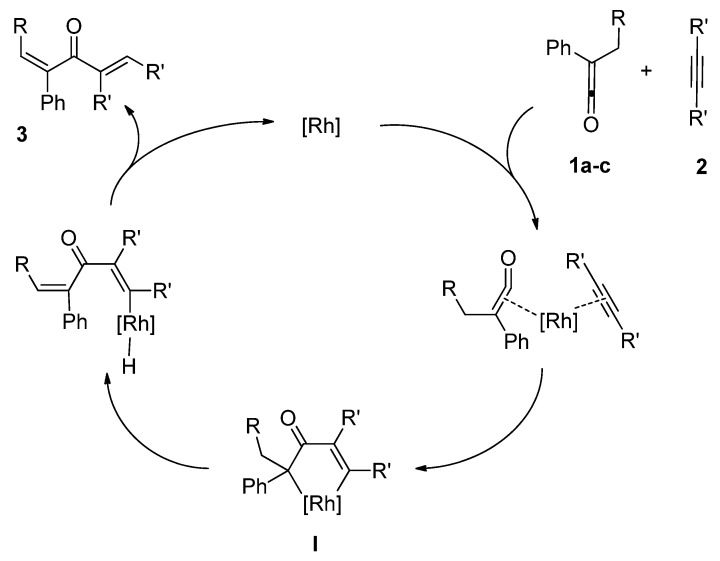
A possible mechanism of linear codimerization of *alkyl phenyl ketenes ***1a**-**c** with internal alkynes **2** to give dienones **3**.

**Scheme 4:**
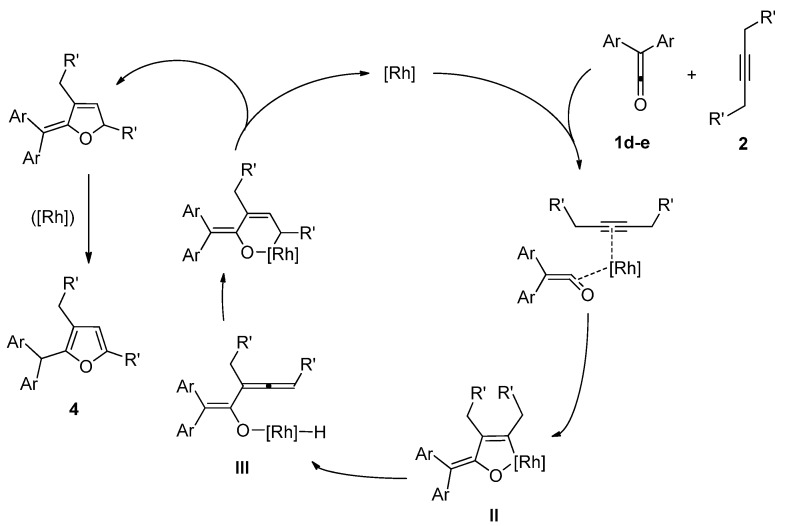
A possible mechanism of the synthesis of furans **4** by unusual cycloaddition of *diaryl ketenes ***1d** and **e** with internal alkynes **2**.

While the reaction mechanism is not yet clear, the possible mechanisms are illustrated in Scheme 3 and Scheme 4. Scheme 3 shows a possible mechanism of the reaction of *alkyl phenyl ketenes ***1a**-**c** with internal alkynes **2** to give dienones **3**. We now believe that the initial step is the coordination of *alkyl phenyl ketenes ***1** to an active rhodium center through a C=C bond in ketenes. Oxidative cyclization of *alkyl phenyl ketenes ***1a**-**c** with alkynes **2** would give rhodacyclopentenone intermediates **I** [5]. Stereoselective *β*-hydrogen elimination, followed by reductive elimination, would give the corresponding dienones **3** stereoselectively. In addition, we now consider that a catalytically active species is a Rh(I) bearing a chloro ligand, and RhCl_3_^.^3H_2_O would be reduced to a Rh(I)-Cl species by crystal water to show some catalytic activity (Entry 4 in Table 1).

On the other hand, a possible mechanism for the reaction of *diaryl ketenes ***1d** and **e** with internal alkynes **2** to furans **4** is shown in Scheme 4. In the synthesis of furans **4**, the reaction starts from the coordination of *diaryl ketenes* to an active rhodium center through a C=O bond in ketenes (not through a C=C bond in ketenes). Oxidative cyclization of *diaryl ketenes ***1d**, and **e** with alkynes **2** gives an oxametallacycle intermediate **II** [22,23,24,25]. *β*-Hydrogen elimination and insertion of an allenyl group in an intermediate **III** into a Rh-H bond, followed by reductive elimination/isomerization, would give the desired furans **4**.

## 3. Experimental

### 3.1. General

GLC analyses were carried out on a Shimadzu GC-18A gas chromatograph equipped with a glass column (2.8 mm i.d. × 3 m) packed with Silicone OV-17 (2% on Chromosorb W(AW-DMCS), 60–80 mesh). Recycling preparative HPLC was performed with an LC-918 (Japan Analytical Industry Co. Ltd.) equipped with JAIGEL-1H and 2H columns (GPC) using CHCl_3_ as an eluent. ^1^H-NMR spectra were recorded at 300 or 400 MHz, and ^13^C-NMR spectra were recorded at 75 or 100 MHz. Samples were analyzed in CDCl_3_, and the chemical shift values are expressed relative to Me_4_Si as an internal standard. IR spectra were obtained on a Nicolet Impact 410 spectrometer. Elemental analyses were performed at the Microanalytical Center of Kyoto University.

### 3.2. Materials

Ketenes were synthesized as described in the literature [26,27]. Alkynes were obtained commercially and purified before use by standard procedures. RhCl_3_^.^·3H_2_O, [RhCl(cod)]_2_, [Cp^*^RhCl_2_]_2_, [RuCl_2_(CO)_3_]_2_, IrCl(CO)(PPh_3_)_2_, and Pd(PPh_3_)_4_ were obtained commercially and used without further purification. RhCl(PPh_3_)_3_ [28], RhCl(CO)(PPh_3_)_2_ [29], RhH(PPh_3_)_4_ [30], RhH(CO)(PPh_3_)_3_ [31], [RhCl(C_2_H_4_)_2_]_2_ [32], RuCl_2_(PPh_3_)_3_ [33], and RuH_2_(PPh_3_)_4_[34] were prepared as described in the literature.

### 3.3. General procedure for the rhodium-catalyzed reaction of ketenes with alkynes to give dienones and furans

A mixture of ketene **1** (1.0 mmol), alkyne **2** (3.0 mmol), RhCl(PPh_3_)_3_ (0.050 mmol), and mesitylene (1.0 mL) was placed in a two-necked 20-mL Pyrex flask equipped with a magnetic stirring bar and a reflux condenser under a flow of argon. The reaction was carried out at 120 ºC for 12 h with stirring. After the reaction mixture was cooled, the products were analyzed by GLC and isolated by Kugelrohr distillation followed by recycling preparative HPLC. 

**Figure 1:**
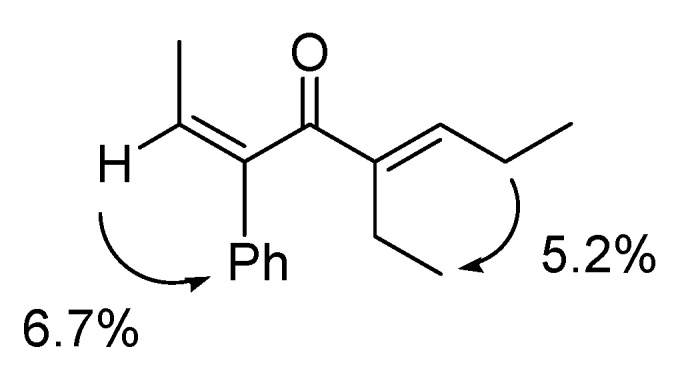
The NOE study of **3a**.

*(2Z,5E)-5-Ethyl-3-phenylocta-2,5-dien-4-one* (**3a**). Yellow liquid; b.p. 130 ºC (3.0 mmHg, Kugelrohr); IR (cm^-1^) 1652 (CO); ^1^H-NMR **(**CDCl_3_, 300 MHz): δ 0.98 (t, 3H, *J *= 7.52 Hz, C8-H), 1.03 (t, 3H, *J* = 7.52 Hz, 5-CH_2_CH_3_), 1.72 (d, 3H, *J* = 7.16 Hz, C1-H), 2.24 (dq, 2H, *J* = 7.52 Hz, C7-H), 2.42 (q, 2H, *J* =7.52 Hz, 5-CH_2_CH_3_), 6.15 (q, 1H, *J* = 7.16 Hz, C2-H), 6.64 (t, 1H, *J* = 7.52 Hz, C6-H), 7.19–7.37 (m, 5H, 3-phenyl-H); ^13^C-NMR (CDCl_3_, 75 MHz): δ 13.09 (5-CH_2_CH_3_), 13.79 (C8), 15.33 (C1), 18.12 (5-CH_2_CH_3_), 22.22 (C7), 125.06 (C2), 125.55 (3-phenyl), 127.25 (3-phenyl), 128.48 (3-phenyl), 129.19 (C3 or C5), 142.85 (3-phenyl-C1), 145.41 (C3 or C5), 150.28 (C6), 200.61 (C4); MS (EI) m/z 228 (M^+^). Anal. Calcd. for C_16_H_20_O: C 84.36, H 9.00. Found: C 84.14, H 8.83. A nuclear Overhauser enhancement (NOE) study was undertaken to determine the stereochemistry of dienone **3a**. Irradiation of olefinic CH at *δ* 6.15 ppm gave a 6.7% NOE of the phenyl group at *δ* 7.26–7.28 ppm, while irradiation of CH_2_ in ethyl group at *δ* 2.24 ppm showed 5.2% NOE with CH_3_ in the other ethyl group at *δ* 1.03 ppm. The stereochemistry of **3a** was therefore assigned to 2*Z* and 5*E* (Figure 1). The same method was used to determine the stereochemistry of **3b**-**d**.

*(2Z,5E)-5-Pentyl-3-phenylundeca-2,5-dien-4-one *(**3b**). Colorless liquid; b.p. 140 ºC (1.0 mmHg, Kugelrohr); IR (cm^-1^): 1652 (CO) ; ^1^H-NMR (CDCl_3_, 400 MHz): δ = 0.84 (t, 3H, C11-H), 0.89 (t, 3H, 5-CH_2_(CH_2_)_3_CH_3_), 1.17–1.35(m, 12H, 5-CH_2_(CH_2_)_3_CH_3_/C8-H/C9-H/C10-H), 1.72 (d, 3H, *J* = 7.16 Hz, C1-H), 2.21 (dt, 2H, C7-H), 2.37 (t, 2H, 5-CH_2_(CH_2_)_3_CH_3_), 6.14 (q, 1H, *J *= 7.16 Hz, C2-H), 6.66 (t, 1H, C6-H), 7.18-7.31 (m, 5H, 3-phenyl-H); ^13^C-NMR (CDCl_3_, 75 MHz): δ 13.93, 14.04, 15.38 (C1), 22.40, 22.51, 24.97, 28.09, 28.61, 29.03, 31.89, 44.87, 125.09 (C2), 125.63 (3-phenyl), 127.14 (3-phenyl), 128.61(3-phenyl), 134.58 (C3 or C5), 141.94 (3-phenyl-1), 144.80 (C3 or C5), 149.70 (C6), 200.62 (C4); MS (EI) m/z 312 (M^+^).

*(3E)-1-Cyclopentylidene-3-ethyl-1-phenylhex-3-en-2-one (**3c**).* Yellow liquid; b.p. 140 ^o^C (2.0 mmHg, Kugelrohr); IR (cm^-1^) 1650 (CO); ^1^H-NMR (CDCl_3_, 400 MHz): δ 0.96 (t, 3H, *J *=7.52 Hz, C6-H), 0.98 (t, 3H, *J = *7.52 Hz, 3-CH_2_CH_3_), 1.61-1.71 (m, 4H, 1-cyclopentylidene-H), 2.21 (q, 2H, *J* = 7.52 Hz, 1-cyclopentylidene-H), 2.30-2.46 (m, 6H, 1-cyclopentylidene-H/C5-H/3-CH_2_CH_3_), 6.63 (t, 1H, *J* = 7.52 Hz, C4-H), 7.17-7.33 (m, 5H, 1-phenyl-H); ^13^C-NMR (CDCl_3_, 100 MHz): δ 13.31 (3-CH_2_CH_3_), 13.77 (C6), 18.51 (3-CH_2_CH_3_), 22.19 (C5), 26.34 (1-cyclopentylidene), 26.38 (1-cyclopentylidene), 32.18 (1-cyclopentylidene), 32.39 (1-cyclopentylidene), 126.50 (1-phenyl), 127.82 (1-phenyl), 128.06 (1-phenyl), 132.91 (1-cyclopentylidene), 138.53 (C1 or C3), 142.08 (1-phenyl-1), 146.26 (C1 or C3), 148.48 (C4), 200.16(C2); MS (EI) m/z 268 (M^+^).

*(3E)-1-Cyclohexylidene-3-ethyl-1-phenylhex-3-en-2-one *(**3d**). Yellow liquid; b.p. 160 ^o^C (8.0 mmHg, Kugelrohr); IR (cm^-1^) 1641 (CO); ^1^H-NMR (CDCl_3_, 400 MHz): δ 0.91 (t, 3H, C6-H), 1.04 (t, 3H, 3-CH_2_CH_3_), 1.60 (br, 6H, 1-cyclohexylidene-H), 2.13 (br, 4H, 1-cyclohexylidene-H), 2.23 (br, 2H, C5-H), 2.31 (q, 2H, *J* = 7.32 Hz, 3-CH_2_CH_3_), 6.78 (t, 1H, *J* = 7.32 Hz, C4-H), 7.21–7.31 (m, 5H, 1-phenyl-H); ^13^C-NMR (CDCl_3_, 100 MHz): δ 13.62 (3-CH_2_CH_3_), 13.79 (C6), 18.46 (3-CH_2_CH_3_), 22.35 (C5), 26.54 (1-cyclohexylidene), 27.96 (1-cyclohexylidene), 28.36 (1-cyclohexylidene), 30.90 (1-cyclohexylidene), 32.88 (1-cyclohexylidene), 126.61 (1-phenyl), 128.77 (1-phenyl), 129.03 (1-phenyl), 133.32 (1-cyclohexylidene), 137.20 (C1 or C3), 140.50 (C1 or C3), 142.81 (1-phenyl-1), 148.96 (C4), 200.32(C2); MS (EI) m/z 282 (M^+^). 

**Figure 2:**
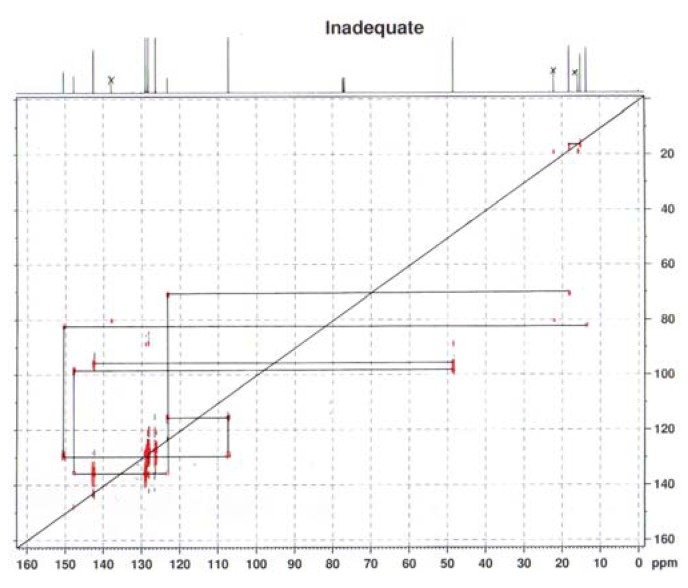
^13^C Inadequate NMR spectrum of **4a**.

*2-(Diphenylmethyl)-3-ethyl-5-methylfuran* (**4a**). Yellow liquid; b.p. 135–145 ºC (0.1 mmHg, Kugelrohr); ^1^H-NMR (CDCl_3_, 400 MHz): δ 1.05 (t, 3H, *J* = 7.57 Hz, 3-CH_2_CH_3_), 2.20 (s, 3H, 5-CH_3_), 2.29 (q, 2H, *J* = 7.49 Hz, 3-CH_2_CH_3_), 5.40 (s, 1H, 2-CHPh_2_), 5.85 (s, 1H, C4-H), 7.18–7.29 (m, 10H, phenyl-H); ^13^C-NMR (CDCl_3_, 100 MHz): δ 13.85 (5-CH_3_), 15.31 (3-CH_2_CH_3_), 18.24 (3-CH_2_CH_3_), 48.43 (2-CHPh_2_), 107.14 (C4), 123.09 (C3), 126.16 (phenyl), 128.24 (phenyl), 128.73 (phenyl), 142.30 (phenyl-1), 147.46 (C2), 150.22 (C5); MS (EI) m/z 276 (M^+^). Anal. Calcd for C_20_H_20_O: C 86.92, H 7.29. Found: C 87.02, H 7.29. The relationship of the substituted group is confirmed by ^13^C Inadequate NMR measurement (Figure 2).

*2-(Diphenylmethyl)-5-n-butyl-3-n-pentylfuran* (**4b**). Yellow liquid; b.p. 170 ºC (0.1 mmHg, Kugelrohr); ^1^H-NMR (CDCl_3_, 400 MHz): δ 0.82 (t, 3H, *J* = 6.84 Hz), 0.89 (t, 3H, *J* = 7.32 Hz), 1.22 (m, 6H, 3-(CH_2_)_2_(CH_2_)_2_CH_3_/5-(CH_2_)_2_CH_2_CH_3_), 1.43 (m, 2H, 3-CH_2_CH_2_(CH_2_)_2_CH_3_), 1.56 (m, 2H, 5-CH_2_CH_2_CH_2_CH_3_), 2.27 (dt, 2H, *J* = 2.12 Hz, 7.69 Hz, 3-CH_2_(CH_2_)_3_CH_3_), 2.53 (t, 2H, *J* = 7.33 Hz, 5-CH_2_(CH_2_)_2_CH_3_), 5.37 (s, 1H, 2-CHPh_2_), 5.82 (s, 1H, C4-H), 7.18–7.29 (m, 10H, phenyl-H); ^13^C-NMR (CDCl_3_, 100 MHz): δ 13.99, 14.16, 22.36, 22.60, 24.97 (3-CH_2_(CH_2_)_3_CH_3_), 27.86 (5-CH_2_(CH_2_)_2_CH_3_), 30.20 (5-CH_2_CH_2_CH_2_CH_3_), 30.34 (3-CH_2_CH_2_(CH_2_)_2_CH_3_), 31.04, 48.38 (2-CHPh_2_), 106.54 (C4), 121.41 (C3), 126.09 (phenyl), 128.01 (phenyl), 128.73 (phenyl), 142.48 (phenyl-1), 147.54 (C2), 154.63 (C5); MS (EI) m/z 360 (M^+^).

*2-(Diphenylmethyl)-5-ethyl-3-n-propylfuran* (**4c**). Yellow liquid; b.p. 140–150 ºC (0.1 mmHg, Kugelrohr); ^1^H-NMR (CDCl_3_, 400 MHz): δ 0.85 (t, 3H, *J* = 7.33 Hz, 3-CH_2_CH_2_CH_3_), 1.17 (t, 3H, *J* = 7.33 Hz, 5-CH_2_CH_3_), 1.47 (sixtet, 2H, *J* = 7.42 Hz, 3-CH_2_CH_2_CH_3_), 2.26 (t, 2H, *J* = 7.57 Hz, 3-CH_2_CH_2_CH_3_), 2.56 (q, 2H, *J* = 7.53 Hz, 5-CH_2_CH_3_), 5.38 (s, 1H, 2-CHPh_2_), 5.83 (s, 1H, C4-H), 7.10–7.29 (m, 10H, phenyl-H); ^13^C-NMR (CDCl_3_, 100 MHz): δ 12.14 (5-CH_2_CH_3_), 14.06 (3-CH_2_CH_2_CH_3_), 21.46 (5-CH_2_CH_3_), 23.79 (3-CH_2_CH_2_CH_3_), 27.08 (3-CH_2_CH_2_CH_3_), 48.36 (2-CHPh_2_), 105.86 (C4), 121.32 (C3), 126.18 (phenyl), 128.11 (phenyl), 128.82 (phenyl), 142.56 (phenyl-1), 147.81 (C2), 156.00 (C5); MS (EI) m/z 304 (M^+^).

*2-(Diphenylmethyl)-3-n-butyl-5-n-propylfuran* (**4d**). Yellow liquid; b.p. 150–160 ºC (0.1 mmHg, Kugelrohr); ^1^H-NMR (CDCl_3_, 300 MHz): δ 0.84 (t, 3H, *J* = 7.25 Hz, 3-CH_2_(CH_2_)_2_CH_3_), 0.91 (t, 3H, *J* = 7.52 Hz, 5-(CH_2_)_2_CH_3_), 1.24 (m, 4H, 3-CH_2_(CH_2_)_2_CH_3_), 1.59 (sixtet, 2H, *J* = 7.38 Hz, 5-CH_2_CH_2_CH_3_), 2.27 (t, 2H, *J* = 7.52 Hz, 3-CH_2_(CH_2_)_2_CH_3_), 2.50 (t, 2H, *J* = 7.43 Hz, 5-CH_2_CH_2_CH_3_), 5.37 (s, 1H, 2-CHPh_2_), 5.83 (s, 1H, C4-H), 7.17-7.27 (m, 10H, phenyl-H); ^13^C-NMR (CDCl_3_, 75 MHz): δ 13.71, 13.89, 21.39 (5-CH_2_CH_2_CH_3_), 22.41, 24.58 (3-CH_2_(CH_2_)_2_CH_3_), 30.10 (5-CH_2_CH_2_CH_3_), 32.72, 48.36 (2-CHPh_2_), 106.79 (C4), 121.52 (C3), 126.23 (phenyl), 128.16 (phenyl), 128.88 (phenyl), 142.67 (phenyl-1), 147.77 (C2), 154.67 (C5); MS (EI) m/z 332 (M^+^). Anal. Calcd for C_24_H_26_O: C 86.70, H 8.43. Found: C 86.43, H 8.67.

*2-[Bis(4-chloropheny)methyl]-3-ethyl-5-methylfuran* (**4e**). Yellow liquid; b.p. 150 ºC (3.0 mmHg, Kugelrohr); ^1^H-NMR (CDCl_3_, 300 MHz): δ 1.05 (t, 3H, *J* = 7.57 Hz, 3-CH_2_CH_3_), 2.20 (s, 3H, 5-CH_3_), 2.28 (q, 2H, *J* = 7.49 Hz, 3-CH_2_CH_3_), 5.31 (s, 1H, 2-CH(*p*-ClPh)_2_), 5.86 (s, 1H, C4-H), 7.06–7.31 (m, 8H, phenyl-H); ^13^C-NMR (CDCl_3_, 75 MHz): δ 13.63 (5-CH_3_), 15.10 (3-CH_2_CH_3_), 18.06 (3-CH_2_CH_3_), 47.08 (2-CH(*p*-ClPh)_2_), 107.35 (C4), 128.47 (C3), 130.13 (phenyl), 132.03 (phenyl), 132.38 (phenyl), 140.56 (phenyl-1), 146.54 (C2), 150.87 (C5); MS (EI) m/z 344 (M^+^).

## 4. Conclusions

In conclusion, we have developed a novel rhodium-catalyzed cross-coupling reaction of ketenes with alkynes. The different coordination modes of ketenes to rhodium, which highly depend on the structure and reactivity of the starting ketenes, realized the selective formation of totally different products, dienones and furans in the presence of the same rhodium catalyst, RhCl(PPh_3_)_3_. Both reactions proceed via characteristic rhodacyclic intermediates.
